# From doubt to doctorate: Navigating imposter syndrome and thriving in physiology‐based grad studies

**DOI:** 10.1113/EP092244

**Published:** 2024-10-18

**Authors:** Joel S. Burma

**Affiliations:** ^1^ Sport Injury Prevention Research Centre, Faculty of Kinesiology University of Calgary Calgary Alberta Canada; ^2^ Cerebrovascular Concussion Laboratory, Faculty of Kinesiology University of Calgary Calgary Alberta Canada; ^3^ Hotchkiss Brain Institute University of Calgary Calgary Alberta Canada

## 1 GRADUATE SCHOOL

Graduate school in Canada is a unique experience varying widely across universities, faculties, and sometimes within the same laboratory. Projects differ in scope, timelines shift unpredictably, and domains of study are individualized to each student's interest. However, a common feeling or perception emerges as a near‐universal phenomenon for graduate students in physiology: imposter syndrome (Feigofsky, 2022). While there are numerous different subtypes (Chandra et al., 2019), imposter syndrome is a belief in which individuals doubt their accomplishments and have a persistent fear of being exposed as a fraud despite evidence of their competence and success. Regardless of your topic of interest, the feeling you do not truly belong, you are not good enough, or that you do not know enough to be offered the opportunity you have paralyses project advancement and personal development. Importantly, this feeling is not achievement‐dependent, with the most accomplished graduate students and early career researchers noting their struggles with this mindset. This was my case, where I thought if I had X number of publications, I would feel competent and knowledgeable in my domain. Yet despite hitting several of these milestones and completing my Doctoral studies with 62 peer‐reviewed publications, the feeling of inadequacy never subsided. Upon deeper reflection and discussions with others, I realized the feeling of being an imposter is intrinsic to being a grad student. We create or lead a project with the guidance of our supervisors, whose primary role is to help foster our development. Further, we receive feedback from a committee (i.e., a panel of physiological experts) to refine and develop our thesis/dissertation, with their novel insight based on their specific field of study. However, the committee is generally composed of numerous experts across different domains who can provide feedback on a unique aspect of a project. This highlights that while grad students may feel insecure because they are dealing with the knowledge of experts, these experts generally are world‐renowned within a very specific domain of research, and deviation into a different domain in the same faculty will leave them feeling like an imposter. Further, an individual is only considered an expert in their field once they have defended their graduate degree. Therefore, rather than feeling negative about imposter syndrome, embrace it and accept that you are not an expert in your topic yet. Use it as motivation to remain curious and ask questions; no one expects you to have all the answers during the trainee stage of your academic journey.

One of the most valuable lessons I learned across my graduate degrees was to reframe rejection and failure as positive. Instead of seeing them as signs of inadequacy, I began to view them as opportunities for growth and development. With every unsuccessful grant, I learned how to adapt my writing to better pitch my project; with every failed experiment, I learned how to troubleshoot on the fly, increasing my adaptability skills; with every journal rejection, I learned resilience and perseverance to modify the manuscript despite past rejection. However, where I grew the most from rejection was through reviewer feedback on submitted manuscripts. While reviewer number two can prove challenging, reviewers are able to provide feedback on projects or topics that may be unfamiliar to you, your supervisor, and other co‐authors. They may recommend statistical analyses or methodological considerations you were unaware of and did not consider. When I first received reviewer feedback as a first‐year master's student, I took these personally and as a direct insult to my intelligence. However, over the years, I learned to desire journal revisions as a way to grow as a scientist and refine my skills. Most of the time, the reviewers are providing suggestions to improve a manuscript, which ultimately will only help the overall impact it will have (Berg et al., 2024). Ultimately, embracing rejection as a catalyst for growth has transformed my approach to research, enabling me to evolve and contribute more meaningfully to my field continually.

Finally, graduate school is more than just earning a degree; it's a transformative experience that challenges you to surpass your perceived limitations. My doctoral project was to develop a novel neuroimaging approach consisting of electroencephalography, functional near‐infrared spectroscopy, and transcranial Doppler ultrasound. Most projects focus on one of these modalities; however, I accepted the challenge of attempting to combine all three to be able to collect high‐quality data in a functionally integrated manner. I had significant doubts about my ability to do this, but rather than focusing on the end goal, I tried to learn one new thing a day. This allowed me to break the final results into thousands of individual steps that, when put together, allowed me to arrive at my envisioned end goal. The ever‐shifting frustrations and difficulties were pivotal in my growth as both a researcher and an individual. Each challenge reinforced my belief that true growth often emerges when you step into the unknown, unsure of your success, yet willing to take the leap. While the fear of failure was a regularly experienced feeling, it paled in comparison to the exhilaration of overcoming the obstacles I experienced. Additionally, when you take that step outside of your comfort zone, even if you do not obtain the results you hoped for, you can be proud of pushing your boundaries. Nevertheless, when you do succeed, when a new method yields results, when your presentation sparks meaningful discussions, or when an interdisciplinary collaboration leads to innovative insights, the sense of accomplishment is unparalleled. These experiences taught me that the most significant progress often arises from embracing discomfort and uncertainty, leaving me more confident and resilient in tackling future challenges.

## 2 DEFENCE DAY

The master's or doctoral defence is often perceived as one of the most daunting hurdles. However, simply viewing it as an examination of one's abilities can place unnecessary stress and anxiety on the individual. It is imperative to remember the role of the examination committee. While these individuals are ensuring you have met the standards required for a graduate degree, they ultimately are on your side and want to see you defend successfully. They will challenge your thinking and get you outside of your knowledge zone, which will help refine and sharpen your ability to formulate an argument concisely. Hence, the defence is not simply a final exam; it is an opportunity to demonstrate how you have grown as a researcher across your degree. The defence is a culmination of years of learning, not just about your specific research topic, but about resilience, critical thinking and open‐mindedness. Moreover, it is important to remain flexible and adaptable during the defence, as one may not know potential unforeseen challenges. With defences being increasingly hybrid or online formats, there is always the risk of technological issues, which was the case for my defence. Rather than allowing this unpredictability to shake my confidence, I embraced it. I understood that rigidity would only limit my ability to effectively communicate my research and engage with my examiners. Therefore, I focused my energy on the things that were in my direct control. Ultimately, the defence is a celebration of everything you have accomplished. It is an opportunity to share your years of hard work with fellow colleagues, friends and family who similarly are there to support you and want to see you succeed.

## 3 CONCLUSION

My journey from imposter to PhD was not a straight path, nor was it free of doubt. But through every challenge, I learned to defeat the doubt by reframing my experiences, stepping outside my comfort zone, and embracing the support of those around me. As I look back on this journey, I realize that it was not just about earning a degree; it was about growing as a researcher, as a scholar and as a person.

## AUTHOR CONTRIBUTIONS

Sole author.

## CONFLICT OF INTEREST

The author declares no conflicts of interest.

## FUNDING INFORMATION

The work in this manuscript was supported by the Natural Sciences and Engineering Research Council (CGSD3‐559333‐2021) and the University of Calgary.
